# Adipose-Derived Mesenchymal Stem Cells Alleviate ᴅ-Galactose-Induced Testicular Injury by Activating the Keap1/Nrf2 Pathway and Suppressing NLRP3-Associated Pyroptosis

**DOI:** 10.3390/antiox15080989

**Published:** 2026-08-10

**Authors:** Mengjia He, Tianhang Yang, Songpo Liu, Dan Zhang, Zhiran Shui, Xianyao Wang, Tao Song, Jun Tan, Qinghong Kong, Jidong Zhang

**Affiliations:** 1 Department of Immunology, Zunyi Medical University, Zunyi 563000, China; 2 Key Laboratory of Cancer Prevention and Treatment of Guizhou Province, Zunyi Medical University, Zunyi 563000, China; 3 Library, Zunyi Medical University, Zunyi 563000, China; 4 Department of Histology and Embryology, Zunyi Medical University, Zunyi 563000, China; 5 Guizhou Provincial College-Based Key Lab for Tumor Prevention and Treatment with Distinctive Medicines, Zunyi Medical University, Zunyi 563000, China; 6 Collaborative Innovation Center of Tissue Damage Repair and Regeneration Medicine, Zunyi Medical University, Zunyi 563000, China

**Keywords:** adipose-derived mesenchymal stem cells, aging-like testicular injury, oxidative stress, Keap1/Nrf2, NLRP3, pyroptosis, Sertoli cells

## Abstract

**Objective:** To evaluate whether human adipose-derived mesenchymal stem cells (ADSCs) protect against ᴅ-galactose (ᴅ-gal)-induced aging-like testicular injury and to investigate the involvement of the Keap1/Nrf2 pathway and NLRP3-associated pyroptosis. **Methods:** A mouse model of aging-like testicular injury was established by subcutaneous administration of ᴅ-gal for 8 weeks, followed by tail vein injection of ADSCs. Testicular morphology, blood–testis barrier (BTB) integrity, and senescence-associated markers (p16, p21) were assessed. In vitro, TM4 Sertoli cells were used to establish a senescence model and Tranwell co-cultured with ADSCs. Oxidative stress, inflammatory responses, and pyroptosis-related markers were evaluated using biochemical assays, immunofluorescence, Western blotting, and RT-qPCR. The involvement of the Keap1/Nrf2-NLRP3 axis was further examined using pharmacological inhibitors. **Results:** ADSC treatment significantly alleviated ᴅ-gal-induced testicular atrophy and histopathological injury, accompanied by reduced expression of the senescence markers p16 and p21 and partial restoration of BTB-related structures. ADSCs also attenuated oxidative stress, as evidenced by decreased ROS and MDA levels, increased SOD activity, and enhanced expression of Nrf2 and its downstream antioxidant targets, including HO-1 and NQO1. In parallel, ADSC administration suppressed NLRP3 activation, reduced caspase-1 cleavage, and lowered the expression of pro-inflammatory cytokines. In TM4 cells, inhibition of Nrf2 weakened the protective effects of ADSCs and was accompanied by reactivation of NLRP3-associated signaling, whereas inhibition of NLRP3 attenuated senescence- and inflammation-related changes without restoring Nrf2 activity. **Conclusions:** ADSCs alleviate ᴅ-gal-induced aging-like testicular injury, at least in part, by restoring redox balance, preserving BTB-associated structure, and suppressing NLRP3-associated pyroptosis through the Keap1/Nrf2 pathway. These findings suggest that ADSC-based therapy may represent a promising strategy for age-related male reproductive dysfunction, while further studies using genetic models and functional fertility endpoints are needed to confirm causality and translational relevance.

## 1. Introduction

In recent years, an increasing number of couples worldwide have chosen to delay childbearing due to educational and career considerations [1], leading to a growing proportion of older fathers [2]. Evidence suggests that signs of male reproductive aging may begin as early as 30 years of age [3]. As the central organ of the male reproductive system, the testis undergoes progressive structural and functional decline with aging, which is associated with reduced fertility and adverse reproductive outcomes [4,5]. These changes have also been linked to increased risks in offspring, highlighting the broader clinical significance of male reproductive aging [6]. Current therapeutic strategies remain limited. Testosterone replacement therapy can partially alleviate symptoms of androgen deficiency; however, exogenous testosterone suppresses the hypothalamic–pituitary–gonadal axis via negative feedback, thereby inhibiting endogenous testosterone production and spermatogenesis [7]. In some cases, this may result in impaired fertility. Assisted reproductive technologies can bypass fertilization barriers but do not restore the testicular microenvironment or repair underlying cellular damage. Therefore, there is a need for novel strategies that can improve the testicular microenvironment and preserve endogenous reproductive function.

Mesenchymal stem cells (MSCs) have been extensively investigated as a promising therapeutic approach for age-related disorders due to their multipotent differentiation capacity, immunomodulatory properties, and tissue repair potential [8]. Among them, human adipose-derived mesenchymal stem cells (ADSCs) offer several practical and biological advantages, including ease of acquisition, high yield, strong proliferative capacity, and low immunogenicity [9]. Importantly, ADSCs exert potent anti-inflammatory and antioxidant effects through paracrine signaling such as extracellular vesicles and specific secretomes, enabling them to modulate the local microenvironment and mitigate oxidative stress-induced damage [10,11,12]. These properties suggest that ADSCs may have therapeutic potential in the context of testicular aging.

Oxidative stress and chronic inflammation are recognized as key drivers of testicular aging, contributing to blood–testis barrier (BTB) disruption, Sertoli cell dysfunction, and impaired spermatogenesis. The aged testis has been characterized as a pro-oxidant and pro-inflammatory microenvironment, while more recent work has highlighted the age-related loss of immune privilege, deterioration of BTB integrity, and functional decline of Sertoli cells [13,14]. Consistent with this framework, experimental evidence has shown that testicular injury can be accompanied by suppression of NRF2 signaling, compromise of BTB-associated structural proteins, and activation of NLRP3-associated inflammatory and pyroptotic pathways [15]. Crucially, Nrf2 typically acts as a negative regulator of the NLRP3 inflammasome by tempering ROS production and suppressing transcription of inflammatory components [16,17]. However, whether ADSCs can alleviate aging-like testicular injury through coordinated regulation of these pathways remains unclear.

ᴅ-galactose (ᴅ-gal) is widely used to model natural aging because it induces persistent oxidative stress and advanced glycation end-product accumulation, and thereby effectively replicates the accelerated oxidative stress and degenerative phenotypes observed in naturally aged tissues [18]. Since Sertoli cells constitute the structural backbone of the blood–testis barrier and are primary targets of age-related oxidative injury [19,20], TM4 cells (an immortalized cell line derived from mouse Sertoli cells) were utilized as a representative in vitro platform. Accordingly, the study investigated the effects of ADSCs on ᴅ-gal-induced aging-like testicular injury in mice and in TM4 Sertoli cells, with a particular focus on the involvement of the Keap1/Nrf2–NLRP3 axis. This work thus establishes a mechanistic link between antioxidant/anti-inflammatory signaling and ADSC-mediated restoration of the aged testicular microenvironment, offering a rational basis for developing stem cell-based therapies for male reproductive aging.

## 2. Materials and Methods

### 2.1. Isolation and Culture of Adipose-Derived Mesenchymal Stem Cells

Human adipose tissue used in this study was obtained from the Affiliated Hospital of Zunyi Medical University, with approval from the Medical Ethics Committee of Zunyi Medical University (Ethics Approval No. [2025] 3-012). Prior to sample collection, all donors (*n* = 3) were screened to exclude infectious diseases, including hepatitis B, syphilis, and HIV. Human adipose-derived mesenchymal stem cells (ADSCs) were isolated from the adipose tissue samples and cultured at 37 °C in a humidified incubator with 5% CO_2_.

To minimize donor-to-donor biological variability and ensure experimental reproducibility, primary ADSCs isolated from the three individual donors were pooled in equal proportions at passage 2 to establish a homogenous cellular population. Cells from this pooled lot at passages 3 to 6 were subsequently utilized for all in vivo and in vitro experiments.

### 2.2. Characterization of ADSCs

Adipogenic and osteogenic differentiation of ADSCs were induced using commercial kits (OriCell, Cyagen Biosciences, Guangzhou, China) according to the manufacturer’s protocols. Briefly, passage 4 cells were seeded in 6-well plates at an appropriate density. When reaching 100% confluence, the growth medium was replaced with OriCell Human ADSC Adipogenic Differentiation Medium (Cat. No. HUXMD-90031) or OriCell Human ADSC Osteogenic Differentiation Medium (Cat. No. HUXMD-90021). The induction medium was renewed every 3 days for 3 weeks. After differentiation, cells were fixed with 4% paraformaldehyde and stained with Oil Red O (OriCell Oil Red O Stain Kit, Cat. No. HUXOR-90011) for lipid droplets or Alizarin Red S (OriCell Alizarin Red S Stain Kit, Cat. No. HUXAR-90011) for calcium deposits, strictly following the kit instructions. Stained cells were observed and imaged under an inverted microscope.

Passage 4 ADSCs were characterized by flow cytometry according to the minimal criteria proposed by the International Society for Cell & Gene Therapy (ISCT). Briefly, cells were harvested using 0.25% trypsin-EDTA, washed twice with PBS containing 1% bovine serum albumin (BSA), and resuspended at a density of approximately 1 × 10^6^ cells/mL. Aliquots of 100 μL cell suspension were incubated with fluorochrome-conjugated monoclonal antibodies against CD73 (PE-Cy7), CD90 (FITC), CD105 (PE), CD34 (APC), CD45 (APC-Cy7), CD11b (APC), and HLA-DR (PE) for 30 min at 4 °C in the dark. After washing twice with PBS, cells were analyzed using a BD FACSCanto II flow cytometer (BD Biosciences, San Jose, CA, USA). At least 10,000 events were acquired for each sample. Data were analyzed using FlowJo software (Version 10.8.1, BD Biosciences/Tree Star). Gates were established according to the corresponding isotype controls. ADSCs were considered to meet the ISCT criteria when they expressed CD73, CD90, and CD105 while lacking expression of CD34, CD45, CD11b, and HLA-DR.

### 2.3. Animals and Experimental Design

Male C57BL/6J mice, aged 6–8 weeks, were provided by the Laboratory Animal Center of Zunyi Medical University (Animal Production License No. SYXK (Qian) 2021-0002). All animal procedures were conducted in accordance with the center’s regulations and were approved by the Institutional Animal Ethics Committee (Ethics Approval No. ZMU21-2412-008). The mice were housed in a specific pathogen-free (SPF) facility under controlled conditions (temperature: 23 °C; 12 h light/dark cycle) with free access to food and water.

Male C57BL/6J mice (6–8 weeks old) were randomly divided into four groups (*n* = 8 per group): a control group, a normal saline (NS) group, a ᴅ-gal group, and a ᴅ-gal + ADSC group. To establish an aging-like model, mice in the ᴅ-gal and ᴅ-gal + ADSC groups were subcutaneously injected with ᴅ-gal (G0750, Sigma-Aldrich, St. Louis, MO, USA) at 300 mg/kg/day for 8 consecutive weeks; this dose was selected based on previous reports demonstrating that it effectively induces accelerated testicular aging and oxidative damage [21,22,23]. The control group received no special treatment, while the NS group received subcutaneous injections of an equal volume of physiological saline over the same 8-week period. After the first 4 weeks of ᴅ-gal administration, the ᴅ-gal-treated mice were randomly subdivided into two subgroups: one subgroup continued to receive ᴅ-gal alone for the remaining 4 weeks (ᴅ-gal group), and the other subgroup received intravenous injections of 1 × 10^6^ adipose-derived stem cells (ADSCs) via the tail vein once weekly for 4 consecutive weeks, with ᴅ-gal administration maintained throughout (ᴅ-gal + ADSC group).

### 2.4. Cell Culture and Transwell Co-Culture Treatment In Vitro

TM4 cells, an immortalized mouse Sertoli cell line, were obtained from Procell (CL-0456, Wuhan, China) and cultured in Dulbecco’s Modified Eagle Medium (DMEM; cat. no. 11965092, Gibco, Thermo Fisher Scientific, Waltham, MA, USA) supplemented with 10% fetal bovine serum (FBS; cat. no. 16000044, Gibco, Thermo Fisher Scientific, Waltham, MA, USA) and 1% penicillin-streptomycin (cat. no. 15140122, Gibco) in a humidified incubator at 37 °C with 5% CO_2_. To establish the in vitro senescence model, TM4 cells were challenged with ᴅ-gal for 48 h. To evaluate the contact-independent paracrine effects of ADSCs on ᴅ-gal-injured Sertoli cells, a non-contact co-culture system was established using Transwell chambers (0.4 μm pore size; Corning, Corning, NY, USA). TM4 cells were seeded in the lower wells of 6-well plates at a density of 1 × 10^5^ cells/well. Following cell adhesion, the cells were exposed to ᴅ-gal for 48 h. Concurrently, ADSCs (passages 3–6) were harvested and seeded into the upper Transwell inserts at a density of 1 × 10^5^ cells/insert.

Subsequently, the Transwell inserts containing ADSCs were placed into the lower wells containing the ᴅ-gal-treated TM4 cells, and the co-culture was maintained for an additional 24 h. The control group (TM4 cells cultured alone without ᴅ-gal or ADSCs) and the ᴅ-gal model group (TM4 cells cultured alone with ᴅ-gal) were processed in parallel using empty Transwell inserts to ensure identical experimental conditions. For signaling pathway intervention assays, specific pharmacological inhibitors, such as the Nrf2 inhibitor ML385 (4 μM; MedChemExpress, Monmouth Junction, NJ, USA) or the NLRP3 inhibitor MCC950 (20 nM; MedChemExpress, Monmouth Junction, NJ, USA), were added to the lower chambers 2 h prior to the introduction of the ADSC inserts. Upon completion of the respective incubation periods, the upper inserts were discarded, and the TM4 cells in the lower chambers were harvested for subsequent biochemical, immunofluorescence, Western blotting, and RT-qPCR molecular analyses.

### 2.5. Behavioral Assessment

To evaluate the potential of ADSCs in alleviating systemic aging phenotypes induced by ᴅ-gal, at the end of the experimental period, the Morris water maze test was performed to assess learning and memory ability. Escape latency during training trials and time spent in the target quadrant during the probe trial were recorded to evaluate spatial cognitive performance.

### 2.6. Determination of Testicular Index

At the end of the 8-week experimental period, all mice were weighed to record their final body weight. Following sacrifice, the bilateral testes, along with the attached epididymides, were carefully harvested and cleared of surrounding adipose and connective tissues. The combined wet weight of the bilateral testes and epididymides was immediately measured on an electronic analytical balance. To assess relative organ atrophy, the testicular index (representing the relative weight of the testes and epididymides) was calculated using the following formula:

$$\mathrm{Testicular}\;\mathrm{Index}\;(g/g)=\frac{\mathrm{Combined}\;\mathrm{Weight}\;\mathrm{of}\;\mathrm{Bilateral}\;\mathrm{Testes}\;\mathrm{and}\;\mathrm{Epididymides}\;(g)}{\mathrm{Final}\;\mathrm{Body}\;\mathrm{Weight}\;(g)}$$

The values were expressed as grams of reproductive tissue per gram of body weight (g/g).

### 2.7. Histopathology

Testicular tissues were harvested, fixed in 4% paraformaldehyde, embedded in paraffin, and sectioned at 5 μm thickness. Sections were stained with hematoxylin and eosin (H&E) for histological evaluation.

### 2.8. Biochemical Analysis

The activities of senescence-associated β-galactosidase (SA-β-gal, U/g protein) (Cat# BC2585) and superoxide dismutase (SOD, U/g protein) (Cat# BC1075), as well as the content of malondialdehyde (MDA, nmol/g protein) (Cat# BC0025), were measured in serum, testicular tissue homogenate and TM4 cells. Measurements were performed using commercially available assay kits (Solarbio Science & Technology Co., Ltd., Beijing, China) following the manufacturer’s instructions, with absorbance read using a microplate reader (Thermo Fisher Scientific, Waltham, MA, USA).

### 2.9. Immunofluorescence

Paraffin sections were rehydrated and washed three times with PBS for 5 min each. The sections were then blocked with 10% normal goat serum for 2 h at room temperature and incubated overnight at 4 °C with appropriately diluted primary antibodies in antibody dilution buffer. The primary antibodies used were Nrf2 (1: 200; AF0639, Affinity Biosciences, Cincinnati, OH, USA), Keap1 (1: 200; HA721525, HUABIO, Hangzhou, China), SOX9 (1: 200; AB5535, Sigma-Aldrich, St. Louis, MO, USA), and Occludin (1: 200; ab216327, Abcam, Cambridge, UK). After washing three times with cold PBS for 5 min each, fluorescence signal amplification and dye labeling were performed using the Absin 3-Color kit (abs50089, Absin, Shanghai, China) according to the manufacturer’s instructions, with incubation for 10 min at room temperature in the dark. Finally, the sections were mounted with SlowFade Gold Antifade Reagent containing DAPI and examined under a fluorescence microscope (Leica, Wetzlar, Germany).

### 2.10. Western Blot Analysis

Western blotting was performed using total protein extracts. Equal amounts of protein were separated on 10% SDS-PAGE gels under reducing conditions and transferred onto PVDF membranes using a wet transfer system at 250 mA for 1 h. Membranes were blocked with rapid blocking solution for 15 min and then incubated overnight at 4 °C with primary antibodies diluted 1: 1000 in Tris-buffered saline containing non-fat milk. The following primary antibodies were used: Occludin (ET1701-76, HUABIO, Hangzhou, China), Connexin 43 (HA721312, HUABIO, Hangzhou, China), Nrf2 (AF0639, Affinity Biosciences, Cincinnati, OH, USA), p16^INK4A^ (AF5484, Affinity Biosciences, Cincinnati, OH, USA), p21^CIP1^ (AF6290, Affinity Biosciences, Cincinnati, OH, USA), NQO1 (T56710, Abmart, Shanghai, China), HO-1 (AF5393, Affinity Biosciences, Cincinnati, OH, USA), Keap1 (HA721525, HUABIO, Hangzhou, China), caspase-3 (DF6879, Affinity Biosciences, Cincinnati, OH, USA), caspase-1 (AG-20B-0042-C100, Adipogen, Liestal, Switzerland), NLRP3 (AG-20B-0014-C100, Adipogen, Liestal, Switzerland), Gasdermin D (HA721144, HUABIO, Hangzhou, China), IL-1β (HA723965, HUABIO, Hangzhou, China) and GAPDH (ab9485, Abcam, Cambridge, UK). After washing with TBS-T, membranes were incubated with HRP-conjugated anti-mouse or anti-rabbit secondary antibodies (7074, Cell Signaling Technology, Danvers, MA, USA) diluted 1: 5000. Protein bands were visualized and quantified using a Bio-Rad ChemiDoc MP Imaging System (Bio-Rad, Hercules, CA, USA).

### 2.11. Real-Time Quantitative PCR (RT-qPCR)

Specific primers for target genes were designed and synthesized by Shanghai Sangon Biological Engineering Co. and reverse transcription reactions and real-time PCR assays were performed according to the manufacturer’s protocols. All reverse transcription reactions, including no-template controls and reverse transcriptase-negative controls, were performed in triplicate using the BIO-RAD CFX96 system (Bio-Rad Laboratories). Relative gene expression was calculated using the comparative threshold cycle (Ct) method. The fold change for genes in the experimental groups was calculated by normalizing their expression levels to the mean expression level of the control group, which was set to 1. The primers used are listed in Table S1.

### 2.12. Statistical Analysis

Data were analyzed using GraphPad Prism 10.0 and are presented as the mean ± SD. Student’s unpaired *t* test was used for comparisons between two groups, and one-way analysis of variance followed by Bonferroni or Newman–Keuls post hoc correction was used for multiple comparisons. A *p* value < 0.05 was considered statistically significant.

## 3. Results

### 3.1. Isolation and Characterization of Human Adipose-Derived Mesenchymal Stem Cells

**Figure 1:**
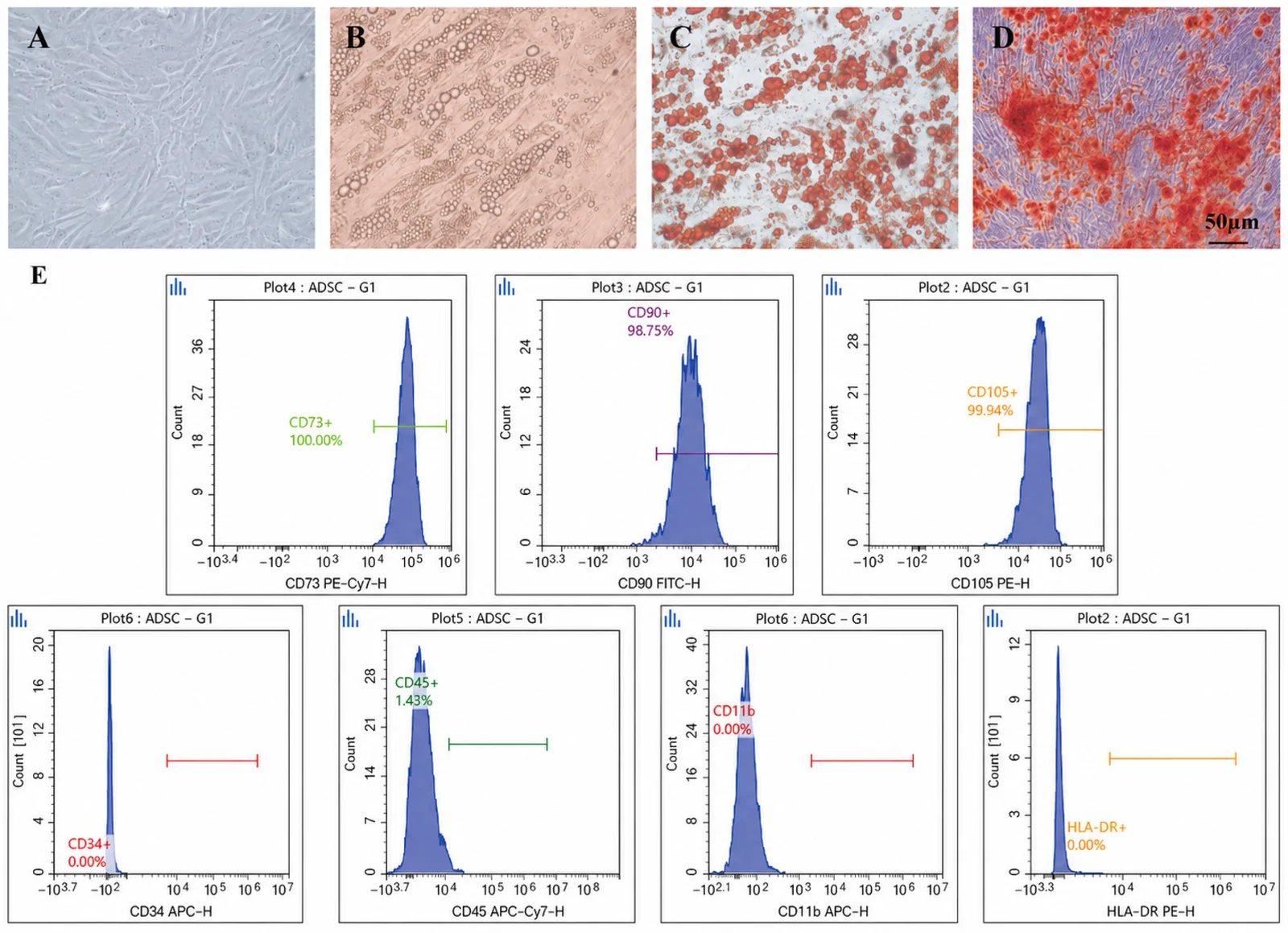
Isolation and identification of ADSCs. (**A**) Representative morphology of ADSCs at passage 4. (**B**,**C**) Adipogenic differentiation of ADSCs, as shown by Oil Red O staining. (**D**) Osteogenic differentiation of ADSCs, as shown by Alizarin Red staining. (**E**) Flow cytometric analysis of ADSC surface markers. ADSCs were positive for CD73, CD90, and CD105, and negative for CD34, CD45, CD11b and HLA-DR.

Following approval from the Ethics Committee (Approval No. [2025] 3-012), human adipose-derived mesenchymal stem cells (ADSCs) were successfully isolated from human abdominal adipose tissue obtained from donors aged 18–30 years and expanded in culture. As shown in Figure 1A, the isolated cells displayed the typical spindle-shaped, fibroblast-like morphology of mesenchymal stem cells. To verify their multipotent differentiation capacity, adipogenic and osteogenic induction assays were performed. Oil Red O staining revealed abundant intracellular lipid droplets after adipogenic induction (Figure 1B,C), whereas Alizarin Red staining demonstrated calcium nodule formation following osteogenic induction (Figure 1D).

Flow cytometric analysis demonstrated that cultured ADSCs strongly expressed the mesenchymal stem cell markers CD73 (100.00%), CD90 (98.75%), and CD105 (99.94%), while showing negligible expression of the hematopoietic and immune-associated markers CD34 (0.00%), CD45 (1.43%), CD11b (0.00%), and HLA-DR (0.00%), consistent with the minimal ISCT criteria for mesenchymal stromal cells (Figure 1E).

### 3.2. ADSCs Alleviate ᴅ-Gal-Induced Aging-like Testicular Injury in Mice

To determine whether ADSCs could attenuate aging-like testicular injury in vivo, a ᴅ-gal-induced aging-like mouse model was established and treated with ADSCs by tail vein injection (Figure S1A). At the end of the experiment, mice in the ᴅ-gal group displayed significant signs of systemic aging phenotype, including dull and rough fur, whereas these phenotypic changes were less pronounced in the ADSC-treated group (Figure S1B), and the Morris water maze test was used to preliminarily examine the systemic senescence of mice induced by ᴅ-gal and the rescue effect of ADSCs: ᴅ-gal-treated mice showed impaired learning and memory performance, as reflected by prolonged escape latency and reduced spatial probe performance, while ADSC treatment partially improved these behavioral outcomes (Figure S1C–E).

**Figure 2:**
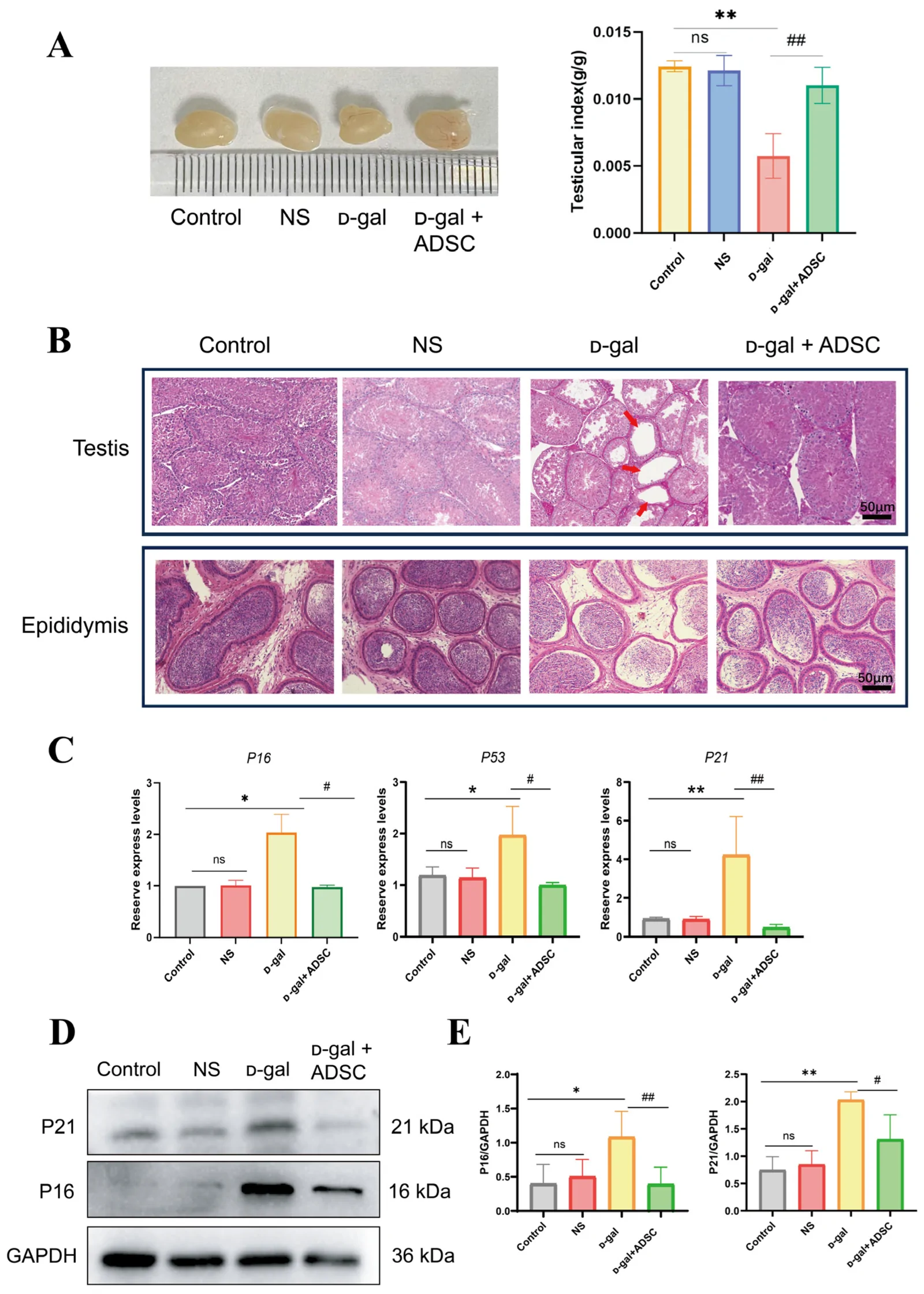
ADSCs alleviate ᴅ-gal-induced aging-like testicular injury in mice. (**A**) Testicular index in each group. (**B**) Representative H&E-stained sections of testicular and epididymal tissues. The sites indicated by the arrows indicate significant vacuolization of the testicular tissue in ᴅ-gal group. (**C**) mRNA expression of senescence-associated genes in testicular tissue. (**D**) Western blot analysis of P16 and P21 in testicular tissue. (**E**) Quantification of P16 and P21 protein expression. Data are shown as indicated. * *p* < 0.05, ** *p* < 0.01 vs. Control; # *p* < 0.05, ## *p* < 0.01 vs. ᴅ-gal; ns, not significant.

One hallmark of the aged testis is a reduction in both volume and weight [24,25]. We next examined testicular morphology and aging-associated markers. The testicular index was significantly reduced in the ᴅ-gal group compared with the control group, whereas ADSC treatment restored the testicular index to a level closer to that of the control group (Figure 2A). Histological analysis by H&E staining showed that the testes of control mice displayed intact seminiferous tubules with orderly arranged germinal epithelium, while ᴅ-gal treatment caused marked testicular atrophy, vacuolar degeneration, and disorganization of the seminiferous epithelium (Figure 2B). These pathological changes were clearly alleviated after ADSC administration. In addition, RT-qPCR and Western blot analyses showed that the senescence-associated markers p16 and p21 were significantly upregulated in the testes of ᴅ-gal-treated mice, whereas ADSC treatment reduced their expression at both the mRNA and protein levels (Figure 2C–E). Together, these results indicate that ADSCs alleviate histopathological damage and decrease the expression of specific senescence-associated markers (p16 and p21) induced by ᴅ-gal, though functional fertility parameters require further validation.

### 3.3. ADSCs Alleviate ᴅ-Gal-Induced Damage to the Testicular Microenvironment and Support Tight Junction Protein Continuity

The blood–testis barrier (BTB) serves as a critical physiological structure for maintaining testicular immune privilege and the stability of the spermatogenic microenvironment [26]. Impairment of BTB integrity resulting from aging can disrupt testicular homeostasis, thereby accelerating the senescence and apoptosis of spermatogenic cells [27]. Given that Sertoli cells constitute the primary cellular component of the BTB and are susceptible to aging-related damage [14], this study focused on investigating the effects of ᴅ-gal on Sertoli cells and the BTB, as well as the interventional effects of ADSCs.

**Figure 3:**
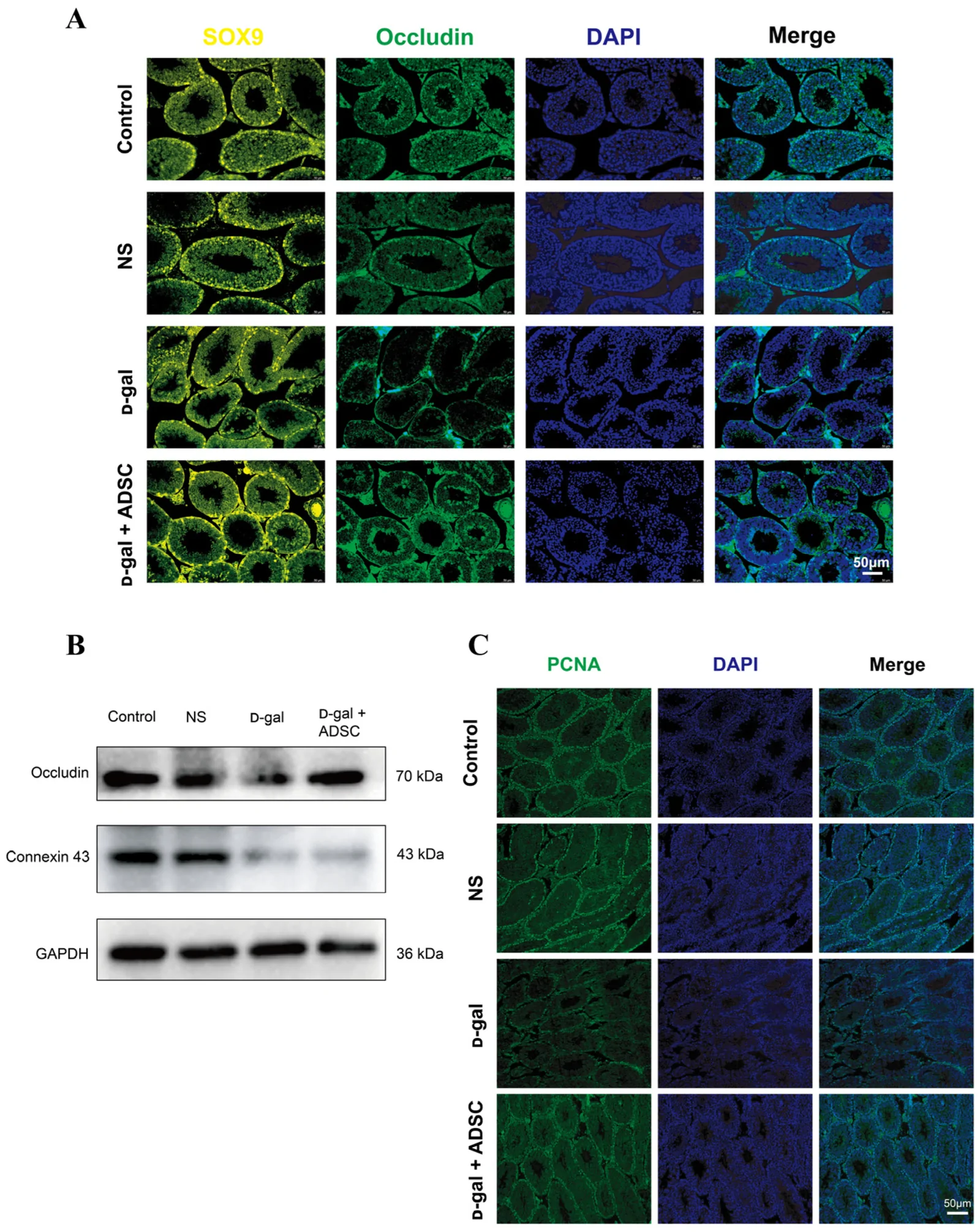
ADSCs improve the spermatogenic microenvironment by preserving blood–testis barrier integrity. (**A**) Immunofluorescence staining of Sertoli cell marker SOX9 (yellow) and the tight-junction protein Occludin (green) in testicular tissue. (**B**) Western blotting of Occludin and Connexin-43 proteins in testicular tissue. (**C**) Immunofluorescence staining of proliferative germ cell-associated marker (PCNA) (green) in testicular tissue.

Immunofluorescence staining showed that SOX9-positive Sertoli cells were markedly reduced in ᴅ-gal-treated mice compared with controls, whereas ADSC treatment increased the number of SOX9-positive cells (Figure 3A). In parallel, Occludin staining, which reflects tight-junction organization at the BTB, appeared discontinuous and disorganized in the ᴅ-gal group, but became more continuous after ADSC intervention. In addition, Western blot results also suggested that the expression of Occludin and Connexin 43 protein in the testis tissue of the mice treated with ᴅ-gal was significantly decreased, which could be effectively restored by ADSC treatment (Figure 3B).

We also assessed spermatogenic niche status by examining PCNA, a proliferative germ cell-associated marker (Figure 3C). Compared with the control group, ᴅ-gal treatment reduced the number of cells with proliferative potential in testicular tissue, suggesting disruption of the spermatogenic microenvironment. ADSC treatment partially restored this population. These findings indicate that ADSCs may help preserve Sertoli cell integrity and BTB-associated organization, thereby protecting the structural basis of the spermatogenic niche in aging testes, to potentially create a more favorable microenvironment for spermatogenesis.

### 3.4. ADSCs Reduce Testicular Inflammation and Mitigate NLRP3-Associated Signaling in ᴅ-Gal-Treated Mice

**Figure 4:**
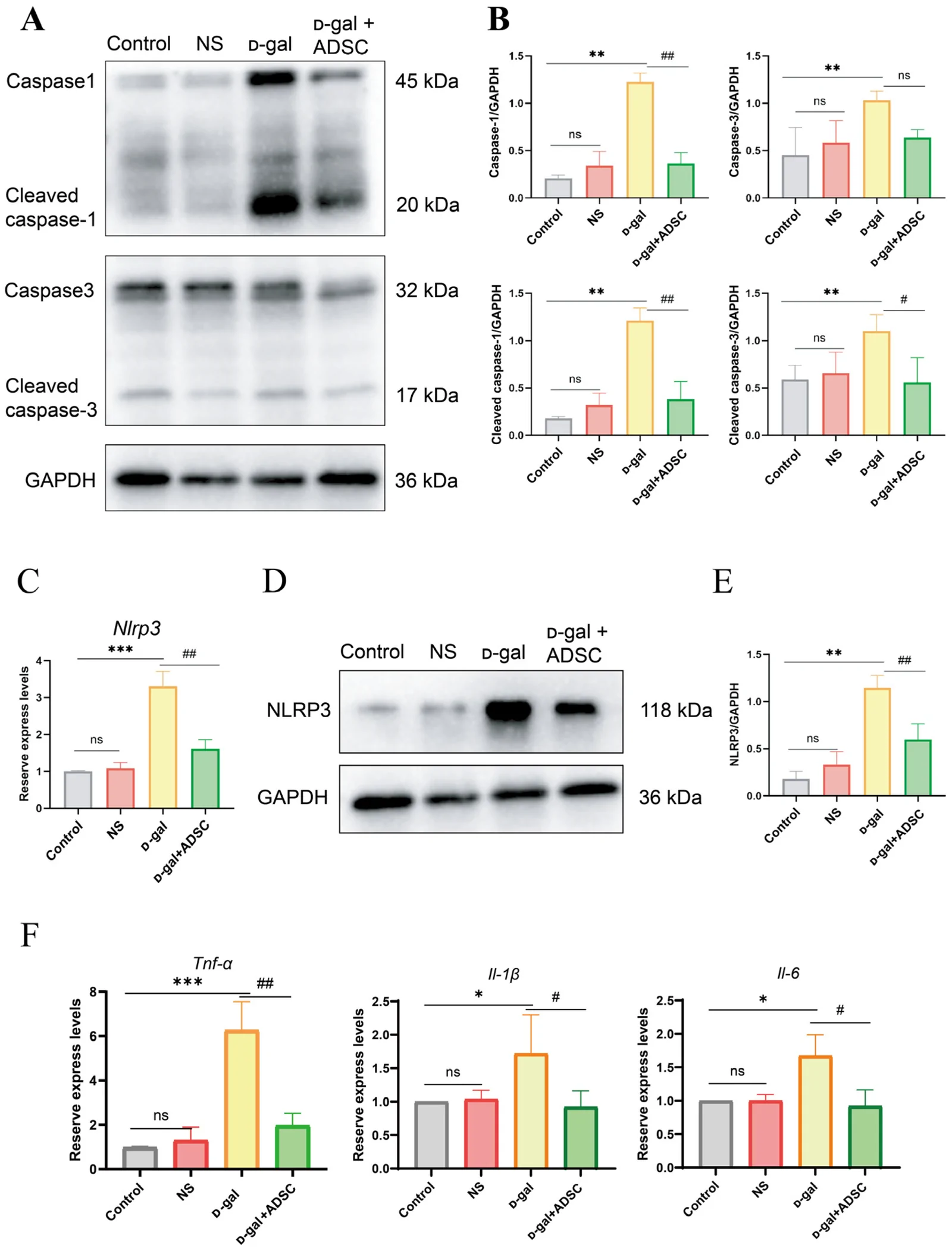
ADSCs reduce inflammation and pyroptosis in ᴅ-gal-treated testes. (**A**) Western blot analysis of caspase-1, cleaved caspase-1, caspase-3, and cleaved caspase-3 in testicular tissue. (**B**) Quantification of caspase-1/caspase-3 and their cleaved forms. (**C**) mRNA expression of Nlrp3 in testicular tissue. (**D**) Western blot analysis of NLRP3 in testicular tissue. (**E**) Quantification of NLRP3 protein expression. (**F**) mRNA expression of inflammatory cytokines *Tnf-α*, *Il-1β*, and *Il-6* in testicular tissue. * *p* < 0.05, ** *p* < 0.01, *** *p* < 0.001 vs. Control; # *p* < 0.05, ## *p* < 0.01 vs. ᴅ-gal; ns, not significant.

Emerging evidence indicates that apoptosis and pyroptosis represent primary modalities of cell death associated with the aging process [28]. To explore the mechanisms underlying testicular cell loss in the aging-like mouse model, we examined key markers associated with these cell death pathways. Western blot analysis showed that ᴅ-gal treatment increased the expression of caspase-1, cleaved caspase-1, caspase-3, and cleaved caspase-3 in testicular tissue, indicating activation of cell death and inflammatory pathways (Figure 4A,B). Following ADSC treatment, the levels of cleaved caspase-1 and cleaved caspase-3 were reduced, whereas total caspase-3 changed only modestly. These collective in vivo data suggest that ᴅ-gal-induced testicular degeneration involves a complex cellular overlay of apoptotic and inflammasome-associated signaling, with ADSCs demonstrating a highly pronounced capacity to suppress the caspase-1-dependent route (Figure 4A,B).

Given that NLRP3 inflammasome assembly serves as the direct upstream canonical trigger for caspase-1 activation, we next quantified NLRP3 expression and its downstream transcriptional mediators. RT-qPCR and Western blot results confirmed that NLRP3 was significantly upregulated in the testes of ᴅ-gal-treated mice, while ADSC administration markedly blunted its expression (Figure 4C–E). In parallel, the mRNA levels of pro-inflammatory cytokines, including IL-β, TNF-α, and IL-6, were heavily elevated in the ᴅ-gal group and were partially normalized after ADSC intervention (Figure 4F). Taken together, these whole-tissue findings indicate that ADSCs effectively remodel the local testicular microenvironment by silencing the NLRP3/caspase-1 inflammatory axis.

Crucially, given that whole-testicular homogenates inherently aggregate cellular signals from a highly heterogeneous niche (including germ cells, Leydig cells, and Sertoli cells), this tissue-level analysis can only capture the thematic activation of the NLRP3 cascade rather than pinpointing its terminal cytolytic execution. Whether this localized activation natively culminates in bona fide, cell-type-specific canonical pyroptosis remains unknown and requires an isolated in vitro somatic cell platform to definitively evaluate the final pore-forming milestones, such as gasdermin D (GSDMD) cleavage.

### 3.5. ADSCs Inhibit Oxidative Stress in the Testes of Aging-like Mice by Activating the Nrf2 Signaling Pathway

**Figure 5:**
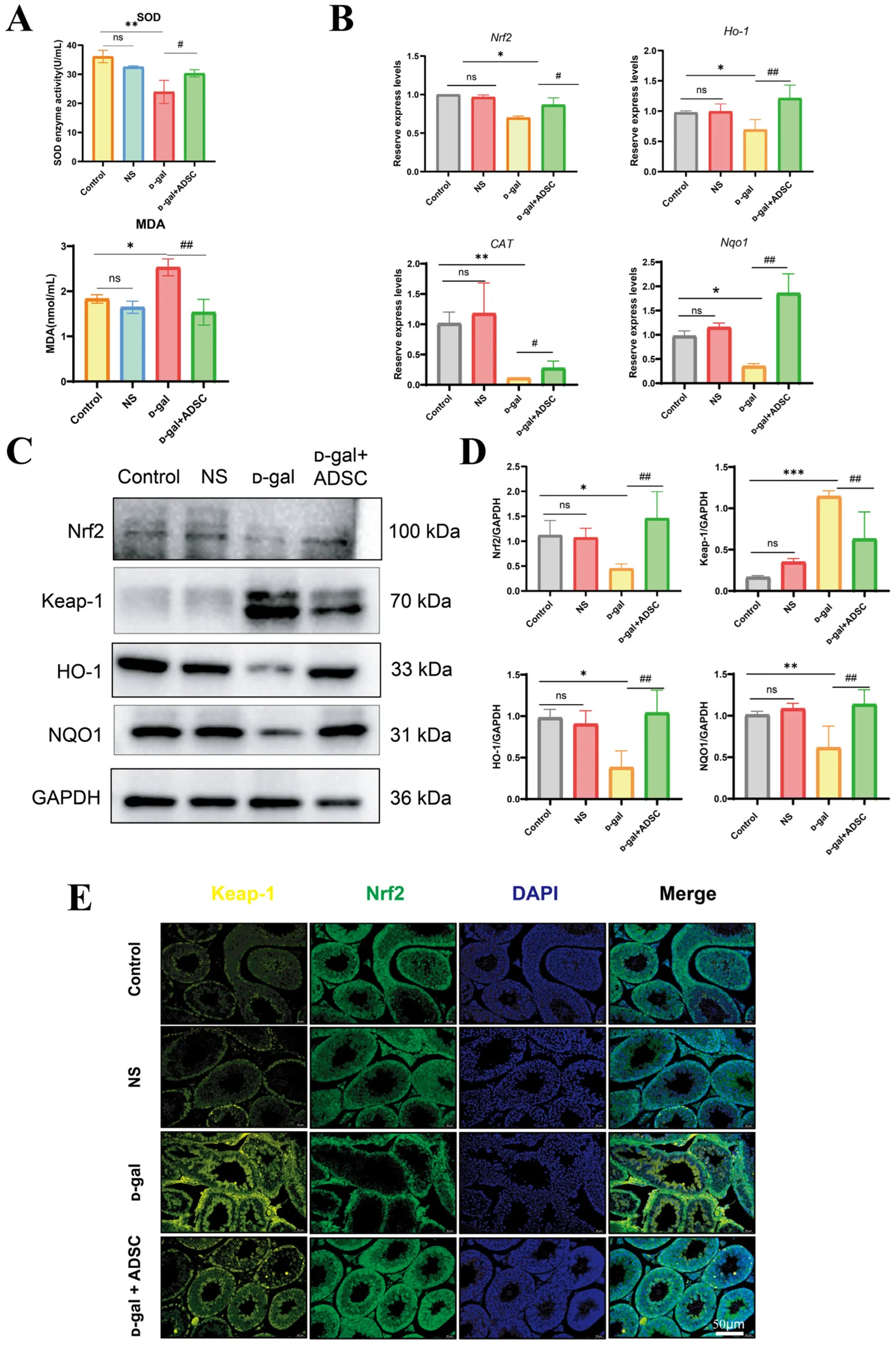
ADSCs attenuate oxidative stress in the testes of ᴅ-gal-treated mice via the Nrf2 pathway. (**A**) Serum SOD activity and MDA content in each group. (**B**) mRNA expression of oxidative stress-related genes in testicular tissue. (**C**) Western blot analysis of Nrf2, Keap1, HO-1, and NQO1 in testicular tissue. (**D**) Quantification of Nrf2, Keap-1, HO-1, and NQO1 protein expression. (**E**) Immunofluorescence staining of Nrf2 (green) and Keap-1 (yellow) in testicular tissue. * *p* < 0.05, ** *p* < 0.01, *** *p* < 0.001 vs. Control; # *p* < 0.05, ## *p* < 0.01 vs. ᴅ-gal; ns, not significant.

Given that the persistent oxidative stress serves as a primary upstream trigger that ignites NLRP3 inflammasome activation and cascades tissue aging [29,30], decoding the local redox status is essential for understanding the microenvironmental restoration mediated by ADSCs. We next assessed systemic redox changes and testicular antioxidant signaling. Biochemical analysis showed that serum SOD activity was significantly reduced, whereas MDA content was increased in ᴅ-gal-treated mice, indicating enhanced oxidative stress; both changes were reversed to varying degrees by ADSC treatment (Figure 5A). These results suggest that ADSCs improve oxidative balance in the aging-like model.

To further define the antioxidant mechanism, we examined the Keap1/Nrf2 axis and its downstream targets in testicular tissue. RT-qPCR and Western blot analyses showed that ᴅ-gal treatment decreased the expression of Nrf2, NQO1, CAT, and HO-1 while increasing Keap1 expression (Figure 5B–D). ADSC treatment partially restored Nrf2 and its downstream antioxidant genes and reduced Keap1 levels. Immunofluorescence staining also showed altered distribution of Nrf2 and Keap1 in testicular tissue after ᴅ-gal treatment, which was partially corrected after ADSC intervention (Figure 5E). These data indicate that ADSCs may protect aging-like testes by enhancing Nrf2-associated antioxidant defense.

Because oxidative stress is predominantly localized within Sertoli cells, subsequent investigations will focus on Sertoli cells as the core candidate target cells mediating the therapeutic effects of ADSCs.

### 3.6. ADSCs Ameliorate ᴅ-Gal-Induced Aging-like Injury and Oxidative Damage in TM4 Cells

**Figure 6:**
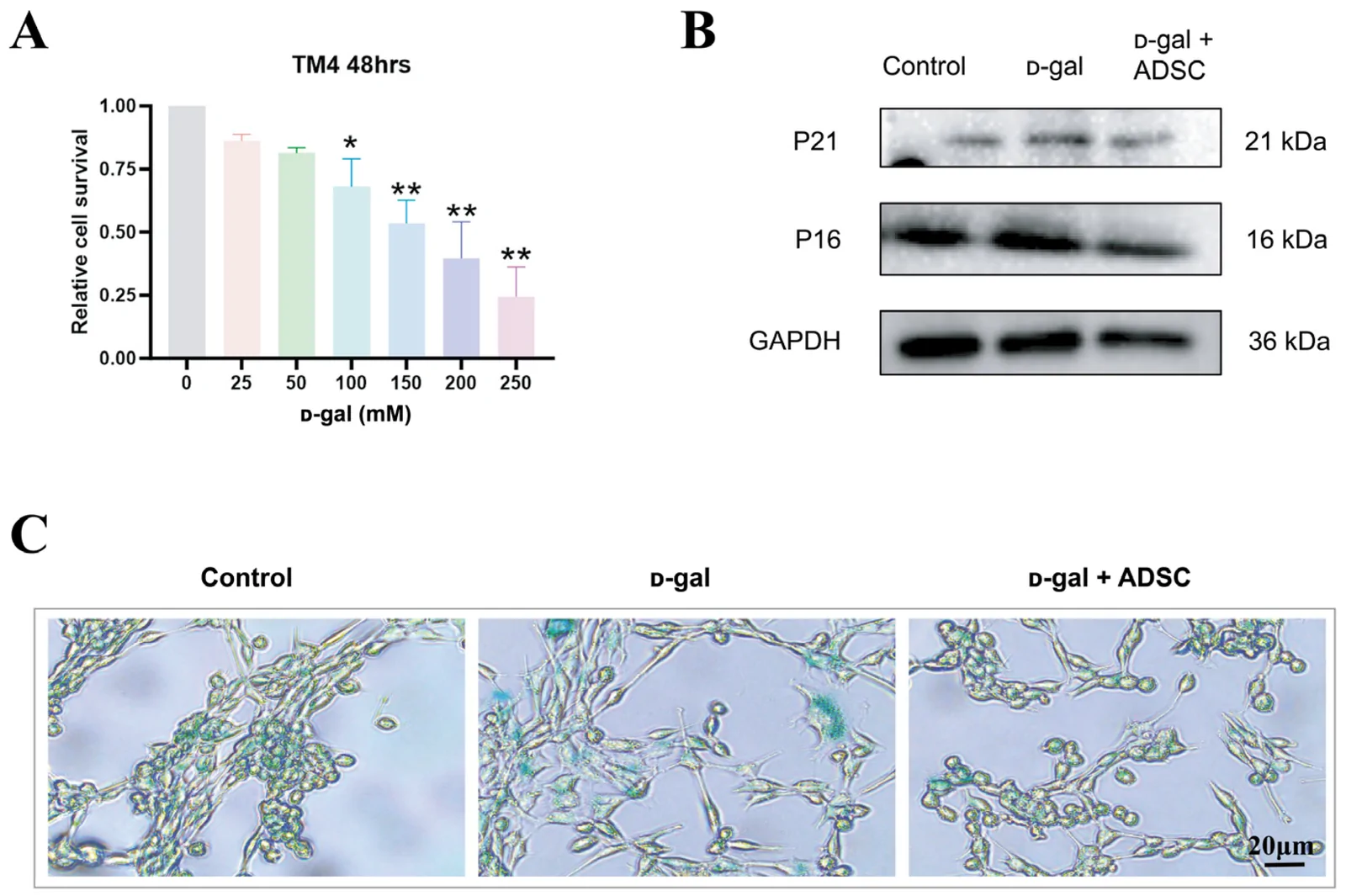
ADSCs alleviate ᴅ-gal-induced aging-like injury in TM4 Sertoli cells. (**A**) CCK-8 assay showing the effect of ᴅ-gal on TM4 cell viability. (**B**) Western blot analysis of P16 and P21 in TM4 cells. (**C**) Representative SA-β-gal staining of TM4 cells after treatment. * *p* < 0.05, ** *p* < 0.001.

TM4 cells are commonly employed as an in vitro model for investigating the spermatogenic microenvironment. To determine whether Sertoli cells are directly affected by ᴅ-gal and whether ADSCs can protect them in vitro, a non-contact Transwell co-culture system was employed to evaluate the paracrine effects of ADSCs on the TM4 senescence model. CCK-8 assay showed that ᴅ-gal reduced TM4 cell viability in a concentration-dependent manner, and 100 mM was selected as the working concentration for subsequent experiments based on preliminary dose–response screening (Figure 6A). The successful establishment of the in vitro aging-like model at this concentration was preliminarily confirmed by SA-β-gal staining (Figure S2A) and measurement of MDA content (Figure S2B). Western blot analyses showed that the senescence-associated markers p16 and p21 were significantly upregulated in ᴅ-gal-treated TM4 cells, whereas ADSC treatment reduced their expression at the protein levels (Figure 6B). In addition, SA-β-gal staining confirmed that ᴅ-gal induced pronounced cellular senescence, whereas ADSC treatment reduced the proportion of SA-β-gal-positive cells (Figure 6C). These findings indicate that ᴅ-gal induces a senescence-like phenotype in TM4 cells and that ADSCs can partially reverse this effect.

### 3.7. ADSCs Suppress Pyroptosis and Oxidative Stress in ᴅ-Gal-Treated TM4 Cells

**Figure 7:**
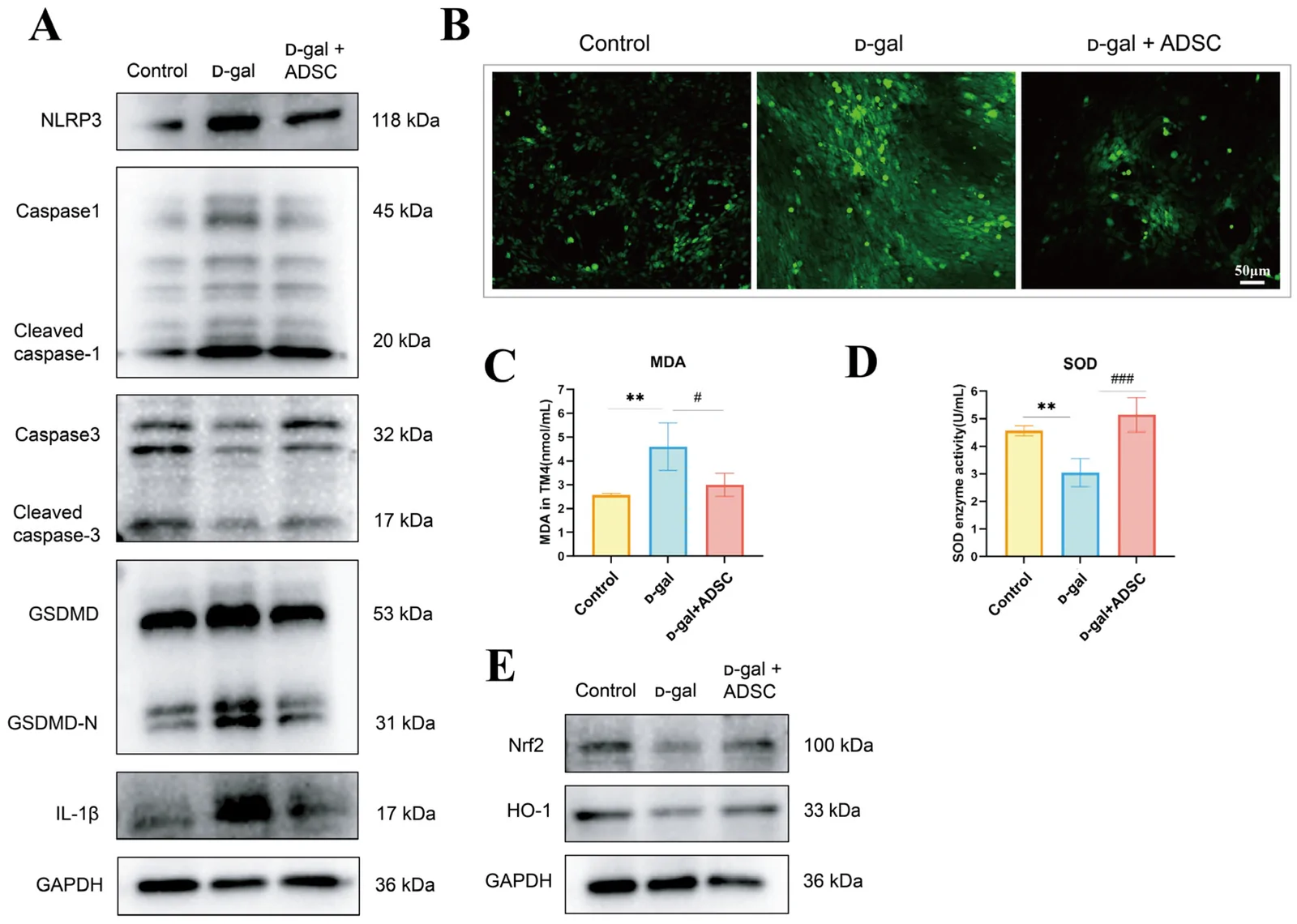
ADSCs suppress inflammation and oxidative stress in ᴅ-gal-treated TM4 Sertoli cells. (**A**) Western blot analysis of caspase-1, cleaved caspase-1, caspase-3, cleaved caspase-3, GSDMD, GSDMD-N, IL-1β and NLRP3 in TM4 cells. (**B**) Intracellular ROS levels assessed by fluorescent probe staining. (**C**) MDA content in TM4 cells. (**D**) SOD activity in TM4 cells. (**E**) Western blot analysis of Nrf2 and HO-1 in TM4 cells. ** *p* < 0.01 vs. Control; # *p* < 0.05, ### *p* < 0.001 vs. ᴅ-gal; ns, not significant.

We then investigated the predominant mode of cell death in TM4 cells under ᴅ-gal stimulation. Western blot analysis showed that ᴅ-gal increased the expression of caspase-1, cleaved caspase-1, and NLRP3, whereas caspase-3 and cleaved caspase-3 were not markedly changed (Figure 7A). Crucially, to definitively verify the activation of canonical pyroptosis, we examined the specific cleavage of GSDMD and the maturation of IL-1β (Figure 7A). The results revealed that ᴅ-gal challenge triggered a striking accumulation of the executive pyroptotic fragment GSDMD-N and mature IL-1β, while total GSDMD levels exhibited only minor variations (Figure 7A). These data firmly demonstrate that canonical pyroptosis, rather than apoptosis, is prominently activated in ᴅ-gal-treated TM4 cells. Notably, Transwell co-culture with ADSCs significantly reduced the expression of pyroptosis-related proteins, indicating that ADSCs exert a protective effect on Sertoli cells via contact-independent paracrine/secretome-mediated effects.

Consistent with the in vivo findings, ᴅ-gal also increased intracellular ROS production and MDA content while reducing SOD activity in TM4 cells, and these changes were partially reversed by ADSC treatment (Figure 7B–D). In addition, ᴅ-gal reduced the expression of Nrf2 and HO-1, whereas ADSCs restored their expression (Figure 7E). Taken together, these results indicate that ADSCs mitigate ᴅ-gal-induced senescence and oxidative injury in Sertoli cells, at least in part, through regulation of antioxidant and pyroptosis-associated pathways.

### 3.8. ADSCs Exert Protective Effects Through the Keap1/Nrf2-NLRP3 Axis

**Figure 8:**
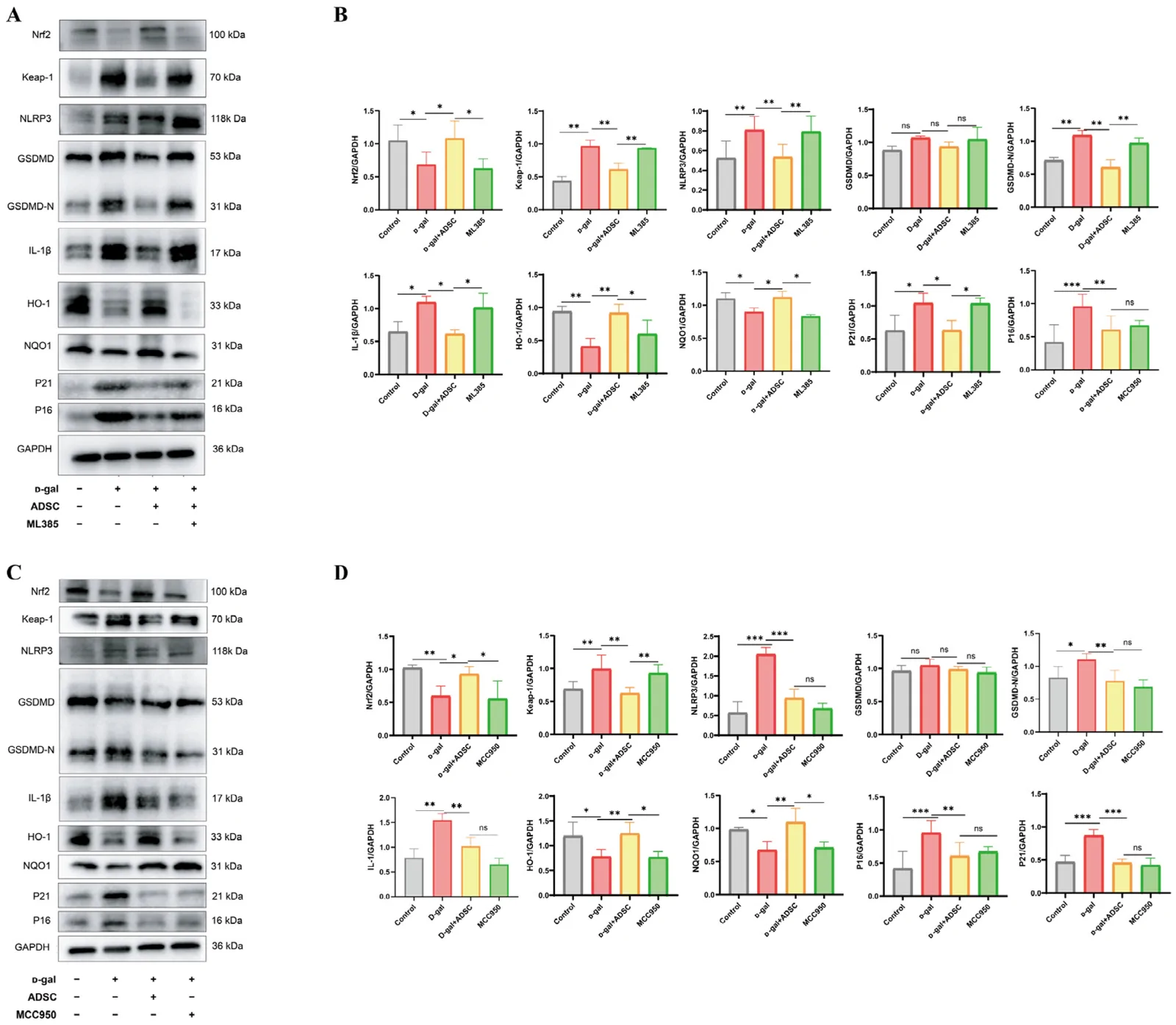
Pharmacological inhibition of Nrf2 or NLRP3 alters ADSC-mediated protection in ᴅ-gal-treated TM4 cells. (**A**) Western blot analysis of Nrf2, Keap-1, HO-1, NQO1, NLRP3, GSDMD, GSDMD-N, IL-1β, P16, and P21 in TM4 cells after ML385 treatment. (**B**) Quantification of the indicated proteins in ML385-treated TM4 cells. (**C**) Western blot analysis of Nrf2, Keap1, HO-1, NQO1, NLRP3, GSDMD, GSDMD-N, IL-1β, P16, and P21 in TM4 cells after MCC950 treatment. (**D**) Quantification of the indicated proteins in MCC950-treated TM4 cells. * *p* < 0.05, ** *p* < 0.01, *** *p* < 0.001; ns, not significant.

To further examine the precise role of Nrf2 in ADSC-mediated protection, TM4 cells were pretreated with ML385, a pharmacological inhibitor of Nrf2. ML385 significantly weakened the ability of ADSCs to restore Nrf2 and its downstream antioxidant targets, including HO-1 and NQO1, while effectively reversing the suppressive effects of ADSCs on p16, p21, and NLRP3 expression (Figure 8A,B). Crucially, the attenuation of terminal pyroptotic executioners by ADSCs was thoroughly neutralized by Nrf2 inhibition. As shown by the quantitative analysis, the ML385-pretreated group exhibited a striking rebound in both the executive pyroptotic fragment GSDMD-N and mature IL-1β protein levels compared to the ADSC-treatment-alone group, whereas total GSDMD levels remained relatively constant (Figure 8A,B). These results firmly demonstrate that ADSCs rely on Nrf2 activation to silence downstream pyroptotic events.

Notably, concomitant with the chemical suppression of Nrf2 expression, the expression level of NLRP3 was significantly elevated (Figure 8A,B). This phenomenon suggests that inactivation of the Nrf2 pathway leads to exacerbated intracellular oxidative stress, which subsequently acts as an uncontrolled upstream driver to passively trigger the downstream NLRP3-mediated cascade.

To definitively map the hierarchical crosstalk between Nrf2 and NLRP3 signaling, the specific NLRP3 inhibitor MCC950 was employed (Figure 8C,D). MCC950 administration efficiently down-regulated NLRP3 expression, attenuated the ᴅ-gal-induced increases in p16 and p21, and robustly suppressed the final cleavage of GSDMD into GSDMD-N as well as the maturation of IL-1β (Figure 8C,D). However, direct pharmacological blockade of NLRP3 failed to rescue the expression of upstream antioxidant elements, including Nrf2, HO-1, and NQO1, nor did it mitigate the over-expression of Keap1 (Figure 8C,D). These compelling findings demonstrate that NLRP3 and its downstream pyroptotic terminal substrates operate as downstream effectors of the Keap1/Nrf2 axis in this model. While halting the NLRP3 hub is sufficient to arrest terminal pyroptotic execution and alleviate specific cellular senescence-related changes, it cannot restore the upstream master antioxidant defense network.

Overall, these data validate a rigorous mechanistic model in which ADSCs protect aging-like Sertoli cells through targeted regulation of the cohesive Keap1/Nrf2–NLRP3 axis.

## 4. Discussion

Male reproductive aging is a complex, multifactorial degenerative process. Unlike the abrupt hormonal transition seen in female menopause, male reproductive decline manifests as a gradual loss of testicular function [13]. While traditional testosterone replacement therapy provides some benefits, its long-term use can suppress the HPG axis, posing significant health risks [31]. Therefore, regenerative medicine strategies aimed at maintaining testicular microenvironmental homeostasis are of substantial clinical value. Due to their accessibility, robust proliferation, low immunogenicity, and potent paracrine properties [32,33], ADSCs have shown great potential across various age-related diseases [34,35]. In the present study, our principal finding was that ADSCs significantly ameliorated ᴅ-gal-induced histopathological damage in mice, downregulated the expression of senescence markers, improved Sertoli cell oxidative-stress homeostasis, and restored the structural integrity of the BTB.

A core manifestation of the aging testis is the disruption of BTB integrity [36]. Although previous studies have observed damage to the spermatogenic niche, they often lacked molecular-level analyses of the BTB’s structural framework [37,38], and our findings provide additional evidence linking Sertoli-cell-associated BTB disruption with coordinated regulation of Nrf2 and NLRP3 signaling. As the primary cellular components of the BTB, Sertoli cells are highly susceptible to age-related injury [39]. In this study, immunofluorescence revealed that ᴅ-gal treatment markedly reduced SOX9-positive Sertoli cells and caused pronounced loosening and fragmentation of the core junctional protein Occludin. ADSC intervention effectively reversed these changes, increasing Sertoli cell counts and promoting the reconnection of Occludin. These findings suggest that the protective effects of ADSCs are not merely generalized tissue repair but involve the precise preservation of Sertoli cell structural integrity to stabilize the spermatogenic microenvironment.

Another critical finding is the activation of canonical pyroptosis signaling following ᴅ-gal-induced testicular injury. Our results show that ᴅ-gal significantly upregulated the expression of the NLRP3 inflammasome and caspase-1. While apoptosis markers were also altered in vivo, pyroptosis activation was more prominent in the TM4 Sertoli cell model, suggesting that NLRP3-mediated pyroptosis may be the primary driver of ᴅ-gal-induced Sertoli cell loss. This cell-type-specific observation is novel, as most prior reports on testicular aging have focused on germ cell apoptosis rather than pyroptosis in somatic Sertoli cells [13,40]. ADSC treatment markedly inhibited NLRP3 expression and reduced the release of pro-inflammatory cytokines like IL-1β. Given that NLRP3 is a highly sensitive sensor of damage signals within the innate immune system [41,42], its downregulation demonstrates that ADSCs can exert therapeutic effects by remodeling the local inflammatory microenvironment. Our study provides the crucial evidence that ADSCs rescue Sertoli cell loss in testicular aging by directly inhibiting NLRP3-mediated pyroptosis, rather than via general anti-inflammation—a mechanism unexplored in this context.

**Figure 9:**
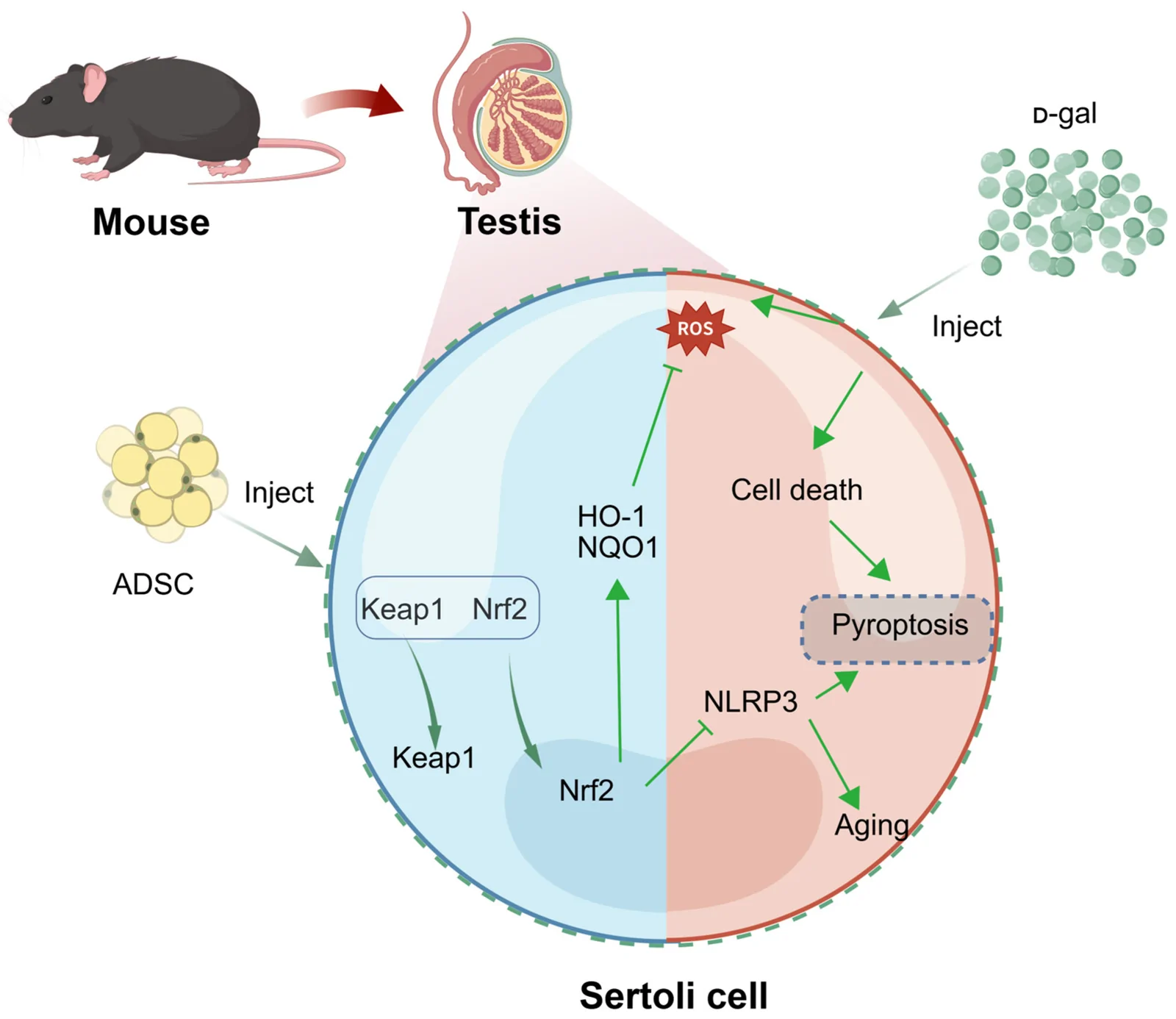
Proposed mechanism by which ADSCs alleviate ᴅ-gal-induced Sertoli cell injury via the Keap1/Nrf2-NLRP3 axis. ADSCs activate Nrf2 signaling in Sertoli cells, promoting nuclear translocation of Nrf2 and inducing downstream antioxidant enzymes, including HO-1 and NQO1, to reduce ᴅ-gal-induced ROS accumulation. In parallel, Nrf2 suppresses NLRP3 inflammasome activation, thereby reducing pyroptosis and senescence and preserving Sertoli cell function (Created with BioGDP.com [52]).

Oxidative stress is a central driver of tissue aging [29,30], and the testis is particularly vulnerable due to its high metabolic activity. This study indicates that ᴅ-gal-induced senescence is accompanied by suppression of the Keap1/Nrf2 antioxidant pathway, characterized by elevated Keap1 and significant decreases in Nrf2, HO-1, and NQO1. ADSCs effectively upregulated the Nrf2-mediated antioxidant defense system, decreasing ROS and MDA levels while restoring SOD activity. This suggests that enhancing redox defense is fundamental to the protective mechanism of ADSCs. Rescue experiments using ML385 (Nrf2 inhibitor) and MCC950 (NLRP3 inhibitor) further supported the molecular relationship: Nrf2 inhibition diminished ADSC-mediated protection and triggered NLRP3 re-elevation, whereas NLRP3 inhibition provided anti-aging benefits without restoring Nrf2 signaling. This suggests that NLRP3 functions downstream of Nrf2, establishing a robust “ADSC–Nrf2–NLRP3–pyroptosis inhibition” axis (Figure 9), which provides a more granular understanding of the crosstalk between redox homeostasis and inflammasome activation compared to previous reports focusing on general cytokine profiles [43].

While the present study provides significant evidence that helps bridge existing gaps in our mechanistic understanding of ADSC-mediated testicular protection [44], several limitations should be acknowledged to guide future translational exploration. Model and Genetic Validation: First, the ᴅ-gal paradigm in this study should be interpreted as an accelerated aging and oxidative stress-associated model rather than a complete recapitulation of physiological, natural testicular aging [45,46]. Furthermore, although ADSCs possess relatively low immunogenicity that supports their frequent use in preclinical transplantation settings [47], the human ADSC/C57BL/6J combination here serves strictly as a proof-of-concept model rather than a direct clinical extrapolation [48]. Mechanistically, our proposed Keap1/Nrf2-NLRP3 axis relies primarily on pharmacological rescue experiments; therefore, future validation using genetic gain- and loss-of-function models will be valuable to definitively map the fine-tuned hierarchical network within this signaling cascade. Functional Endpoints and In Vivo Tracking: Second, this work focused predominantly on the structural restoration of the spermatogenic microenvironment and BTB integrity, lacking a definitive demonstration of functional fertility rescue. Subsequent longitudinal studies incorporating functional fertility endpoints—such as sperm kinetic parameters, mating performance, and offspring outcomes—are essential to complement these molecular findings. Additionally, because the direct homing of transplanted ADSCs to the testes in vivo remains unverified, our conclusions lean heavily on in vitro contact-independent paracrine evidence. Future validation using in vivo cell tracking, syngeneic models, or longitudinal assessments of their homeostatic interactions with healthy, non-injured testicular tissues will provide deeper insights into tissue specificity. Alternative Mechanisms and Translational Optimization: Finally, ADSC-mediated protection likely involves multifaceted layers beyond redox–pyroptosis cross-talk, including secretome-derived exosomal RNAs, mitochondrial stabilization, and anti-apoptotic signaling, which warrant direct assessment. Crucially, overcoming clinical challenges regarding the long-term survival and homing efficiency of transplanted cells within the aged microenvironment remains a primary hurdle [44,49]. Future translational strategies combining ADSCs with advanced biomaterials [50] or protective pretreatments [51] may further optimize the therapeutic performance of stem cell-based interventions for age-related male reproductive dysfunction.

In summary, this study provides evidence that ADSCs alleviate ᴅ-gal-induced aging-like testicular injury by preserving BTB-related structures, reducing oxidative stress and inflammatory injury, and modulating Keap1/Nrf2 signaling and NLRP3-associated pyroptosis. The present findings expand the understanding of how ADSCs may act on the aging testis and support further investigation of stem cell-based strategies for age-related male reproductive dysfunction. At the same time, additional functional and mechanistic studies will be necessary to validate and extend these observations in more clinically relevant settings.

## 5. Conclusions

In summary, the present study demonstrates that ADSCs alleviate ᴅ-gal-induced aging-like testicular injury in mice, as evidenced by improvements in testicular morphology, reduced expression of senescence-associated markers, and partial restoration of BTB-related structures. These protective effects are accompanied by attenuation of oxidative stress and inflammatory responses, as well as reduced expression of pyroptosis-associated molecules.

Mechanistically, our findings are consistent with a model wherein ADSCs enhance antioxidant defenses and suppress inflammation, parallel to the activation of the Keap1/Nrf2 pathway and the downregulation of NLRP3 inflammasome markers. Pharmacological inhibition experiments further support the involvement of these pathways, although additional studies are required to fully establish their causal relationships and upstream regulatory mechanisms.

Overall, this study provides experimental evidence that ADSCs may exert protective effects on the aging testis through coordinated regulation of redox homeostasis and inflammatory signaling. These findings support the potential of ADSC-based approaches for the treatment of age-related testicular dysfunction, while highlighting the need for further validation in functional and translational settings.

## Data Availability

The raw data supporting the conclusions of this article will be made available by the authors on request.

## Supplementary Materials

The following supporting information can be downloaded at: https://www.mdpi.com/article/10.3390/antiox15080989/s1, Figure S1: Effects of ADSCs on ᴅ-gal-induced aging phenotypes in mice; Figure S2: Preliminary verification of the reliability of the model concentration in vitro; Table S1: Sequences of the primer pairs used for real-time PCR.
