# Supplementary material for: Adipose-Derived Mesenchymal Stem Cells Alleviate ᴅ-Galactose-Induced Testicular Injury by Activating the Keap1/Nrf2 Pathway and Suppressing NLRP3-Associated Pyroptosis
Source: Antioxidants (Basel). 2026 Aug 10;15(8):989. doi: 10.3390/antiox15080989 (PMC13509371; doi:10.3390/antiox15080989)
Supplement: Supplementary file 1 [file antioxidants-15-00989-s001.zip › Figure S1 and S2.pdf]

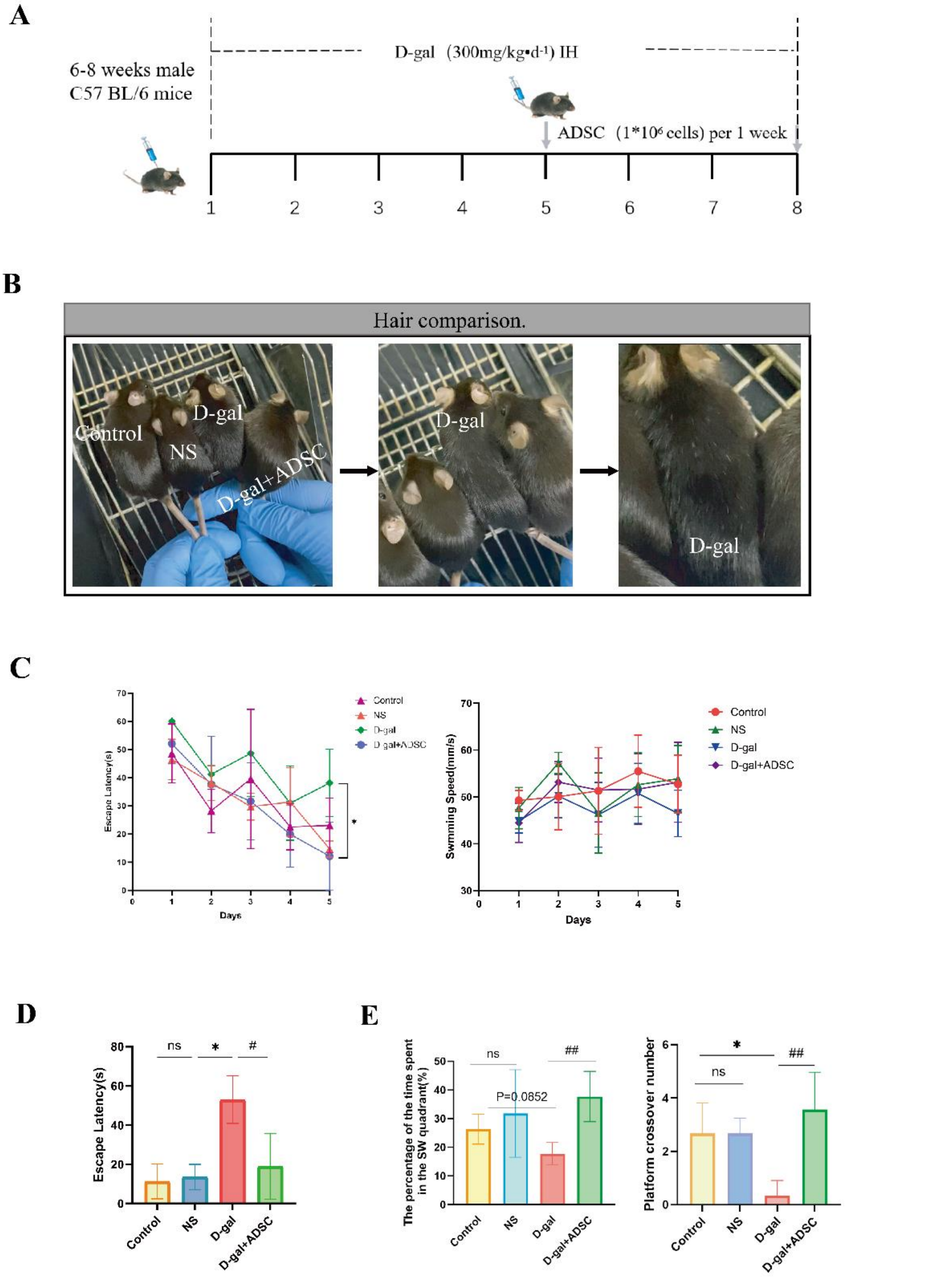

Figure S1. Effects of ADSCs on D-gal-induced aging phenotypes in mice. (A) Schematic illustration of the animal experiment. **(B)** Representative photographs showing the appearance of mice in each group. **(C)** Visible platform trial in the Morris water maze. **(D)** Hidden platform trial in the Morris water maze. **(E)** Probe trial in the Morris water maze. **p* < 0.05, ***p* < 0.01 vs. Control; #*p* < 0.05, ##*p* < 0.01 vs. D-gal; ns, not significant.

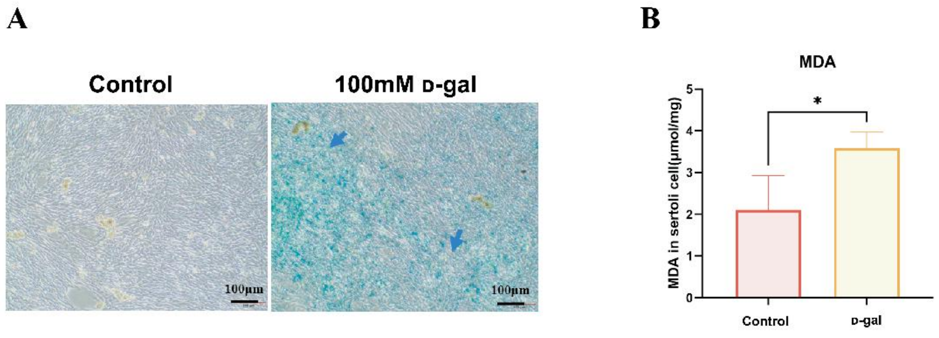

Figure S2. Preliminary verification of the reliability of the model concentration in vitro. (A) Representative SA-β-gal staining of TM4 cells after D-gal treatment. **(B)** MDA content in TM4 cells. *p <0.05.
