# Supplementary material for: Adipose-Derived Mesenchymal Stem Cells Alleviate ᴅ-Galactose-Induced Testicular Injury by Activating the Keap1/Nrf2 Pathway and Suppressing NLRP3-Associated Pyroptosis
Source: Antioxidants (Basel). 2026 Aug 10;15(8):989. doi: 10.3390/antiox15080989 (PMC13509371; doi:10.3390/antiox15080989)
Supplement: Supplementary file 1 [file antioxidants-15-00989-s001.zip › Table S1.pdf]

Table S1. Sequences of the primer pairs used for real-time PCR

Gene	Primer sequence (5'-3')	NCBI RefSeq Accession No.
P16^{INK4A}	Forward CGCAGGTTCTTGGTCACTGT<br>Reverse TGTTCACGAAAGCCAGAGCG	NM_009877
P21^{CIP1}	Forward CCTGGTGATGTCCGACCTG<br>Reverse CCATGAGCGCATCGCAATC	NM_007669
Nlrp3	Forward ATTACCCGCCCGAGAAAGG<br>Reverse TCGCAGCAAAGATCCACACAG	NM_145827
Tnf-α	Forward CCTGTAGCCCACGTCGTAG<br>Reverse GGGAGTAGACAAGGTACAACCC	NM_011601
Il-1β	Forward GCAACTGTTCCTGAACTCAACT<br>Reverse ATCTTTTGGGGTCCGTCAACT	NM_008361
Il-6	Forward TCTATACCACTTCACAAGTCGGA<br>Reverse GAATTGCCATTGCACAACTCTTT	NM_031168
Cat	Forward ATAGCCAGAAGAGAAACCCA<br>Reverse TTCATGTGCCGGTGACCAT	NM_009804
Nqo1	Forward AGGATGGGAGGTACTCGAATC<br>Reverse AGGCGTCCTTCCTTATATGCTA	NM_008706
Keap-1	Forward TGCCCCTGTGGTCAAAGTG<br>Reverse GGTTCGGTTACCGTCCTGC	NM_016679
Ho-1	Forward AAGCCGAGAATGCTGAGTTCA<br>Reverse GCCGTGTAGATATGGTACAAGGA	NM_010442
Nrf2	Forward TCTTGGAGTAAGTCGAGAAGTGT<br>Reverse GTTGAAACTGAGCGAAAAAGGC	NM_010902
